# A novel firmicute protein family related to the actinobacterial resuscitation-promoting factors by non-orthologous domain displacement

**DOI:** 10.1186/1471-2164-6-39

**Published:** 2005-03-17

**Authors:** Adriana Ravagnani, Christopher L Finan, Michael Young

**Affiliations:** 1 Institute of Biological Sciences, University of Wales, Aberystwyth, Ceredigion SY23 3DD, UK

## Abstract

**Background:**

In *Micrococcus luteus *growth and resuscitation from starvation-induced dormancy is controlled by the production of a secreted growth factor. This autocrine resuscitation-promoting factor (Rpf) is the founder member of a family of proteins found throughout and confined to the actinobacteria (high G + C Gram-positive bacteria). The aim of this work was to search for and characterise a cognate gene family in the firmicutes (low G + C Gram-positive bacteria) and obtain information about how they may control bacterial growth and resuscitation.

**Results:**

*In silico *analysis of the accessory domains of the Rpf proteins permitted their classification into several subfamilies. The RpfB subfamily is related to a group of firmicute proteins of unknown function, represented by YabE of *Bacillus subtilis*. The actinobacterial RpfB and firmicute YabE proteins have very similar domain structures and genomic contexts, except that in YabE, the actinobacterial Rpf domain is replaced by another domain, which we have called Sps. Although totally unrelated in both sequence and secondary structure, the Rpf and Sps domains fulfil the same function. We propose that these proteins have undergone "non-orthologous domain displacement", a phenomenon akin to "non-orthologous gene displacement" that has been described previously. Proteins containing the Sps domain are widely distributed throughout the firmicutes and they too fall into a number of distinct subfamilies. Comparative analysis of the accessory domains in the Rpf and Sps proteins, together with their weak similarity to lytic transglycosylases, provide clear evidence that they are muralytic enzymes.

**Conclusions:**

The results indicate that the firmicute Sps proteins and the actinobacterial Rpf proteins are cognate and that they control bacterial culturability via enzymatic modification of the bacterial cell envelope.

## Background

The growth and culturability of the actinobacteria is controlled by a family of secreted or membrane-associated proteins [1]. The Rpf protein of *Micrococcus luteus *was the founder member of this family, which now comprises more than forty representatives [2-4]. Rpf is required for the resuscitation of dormant cells of *M. luteus *and for the growth of sparsely inoculated cultures of this organism in nutrient-poor media. *M. luteus *seems to contain only one *rpf *gene, whose product appears to be essential for bacterial growth [5]. In contrast, most organisms contain several *rpf*-like genes, whose products are functionally redundant [3,6-8]. All the proteins so far tested show cross-species activity in bioassays using laboratory cultures of several different organisms, including *M. luteus*, *Rhodococcus rhodochrous*, *Mycobacterium tuberculosis*, *Mycobacterium bovis *(BCG) and *Mycobacterium smegmatis *[4,7,9,10]. Since they are active at minute concentrations, it was suggested that they might be involved in inter-cellular signalling [1,3,4].

Rpf-like proteins are not found in firmicutes (low G+C Gram-positive bacteria), although some distantly related proteins are found in *Staphylococcus *and *Oenococcus *(see below). In this article we report the results of comparative genomic and domain analyses indicating that the firmicutes contain a cognate protein family related to the actinobacterial Rpf proteins by a process of "non-orthologous domain displacement". The available evidence strongly suggests that both the firmicute and actinobacterial proteins have a catalytic function, which may be responsible for their observed activity in improving the culturability of the organisms that produce them.

## Results

### The Rpf domain

Bacterial genome sequencing projects have uncovered many genes whose products share with *M. luteus *Rpf a ca. 70-residue segment that we have called the Rpf domain. This segment of *M. luteus *Rpf is both necessary and sufficient for biological activity, indicating that it corresponds to a functional protein domain [5]. The Rpf-like proteins appear to be restricted to several genera within the actinobacteria, including *Corynebacterium*, *Micrococcus*, *Mycobacterium*, *Saccharopolyspora *and *Streptomyces*, but they appear to be absent from some others, such as *Bifidobacterium*, *Thermobifida *and *Tropheryma *(Table 1). An alignment of 44 Rpf-like domains revealed that a central region of between 6 and 9 residues accounts for almost all of the observed variation in length of this domain (see additional data file 1). SignalP [11] and TMHMM [12] predictions suggest that all of the Rpf-like gene products so far uncovered are either secreted, or membrane-associated, with the exception of one instance of an Rpf-like domain within a mycobacteriophage tape measure protein [13]. The Rpf domain also contains two highly conserved cysteine residues. Modelling has suggested that they lie in close proximity and may form a disulphide bridge (A. Murzin, personal communication) [14].

**Table 1:** Organisms containing *rpf*-like genes

**Part A**: genes encoding proteins containing a Rpf domain			
Organism	Genome size (Mb)	No. of genes	Genome Accession Number

*Corynebacterium diphtheriae*	2.5	3	NC_002935
*Corynebavterium glutamicum*	3.3	2	NC_003450
*Corynebacterium efficiens*	3.1	2	NC_004369
*Micrococcus luteus*	2.3	1	Mukamolova *et al*, 1998
*Mycobacterium avium*	4.7	4	NC_002944
*Mycobacterium bovis*	4.3	5	NC_002945
*Mycobacterium leprae*	3.3	3	NC_002677
*Mycobacterium marinum*	6.5	4	NC_004506 (unfinished)
*Mycobacterium smegmatis*	7.0	4	NC_002974 (unfinished)
*Mycobacterium tuberculosis *H37Rv	4.4	5	NC_000962
*Streptomyces coelicolor*	8. 7	5	NC_003888
*Streptomyces avermitilis*	9.0	6	NC_003155

**Part B**: genes encoding proteins containing a domain distantly related to the Rpf domain			

*Bifidobacterium longum *NCC2705	2.3	3	NC_004307
*Tropheryma whipplei *strain Twist	0.9	2	NC_004572
*Streptomyces coelicolor*	8. 7	2	NC_003888
*Streptomyces avermitilis*	9.0	3	NC_003155
*Staphylococcus carnosus*	-	2	-
*Staphylococcus aureus *N315	2.8	1	NC_002745
*Staphylococcus epidermidis*	2.6	1	NC_004461
*Oenococcus oeni*	0.3	1	NZ_AABJ02000001

*M. luteus *and *S. carnosus *genomes are not yet sequenced

*M. luteus *genome size taken from Murayama *et al*. [78]

HMMs were used to create profiles of the Rpf domain alignment and these were employed to perform local and global searches of the SWISS-PROT and TrEMBL databases (downloaded from the European Bioinformatics Institute website [15]). In addition to the previously known Rpf domains in the various actinobacterial Rpf-like proteins, which were detected with highly significant *E*-values (5.7·10^-56 ^– 4.8·10^-39^), these searches also identified two *Staphylococcus carnosus *protein precursors, SceD and SceA (054493 and 054494), with much higher, but nevertheless statistically significant *E*-values (7.1·10^-4 ^and 3.9·10^-2^). These proteins contain a domain more distantly related to the Rpf domain. Additional hits above the level of statistical significance (*E*-values more than 0.1) included many c-type lysozyme precursors, which shared similarity with a 24-residue segment towards the C-terminus of the Rpf domain, as has been reported previously [2,14,16]. A PSI-BLAST search was also performed (Blosum62 matrix and a 0.005 *E*-value threshold) using the Rpf domain of *M. luteus *Rpf for the first iteration . No new hits were found after 3 iterations. In addition to the known Rpf-like gene products and the more distantly related SceA & SceD proteins of *S. carnosus*, this search revealed SceD orthologues in two strains of *Staphylococcus aureus *(NP_646837.1 & NP_372619.1; *E*-values 2·10^-3 ^& 3·10^-3^) and *Staphylococcus epidermidis *(NP_765249.1; *E*-value 9·10^-4^) in addition to a previously undetected gene product from *Oenococcus oeni *(ZP_00069230.1; *E*-value 3·10^-13^). These proteins containing a domain distantly related to the Rpf domain are found in the firmicutes, whereas proteins containing the Rpf domain appear to be restricted to the actinobacteria.

### Rpf protein subfamilies

**Figure 1:**
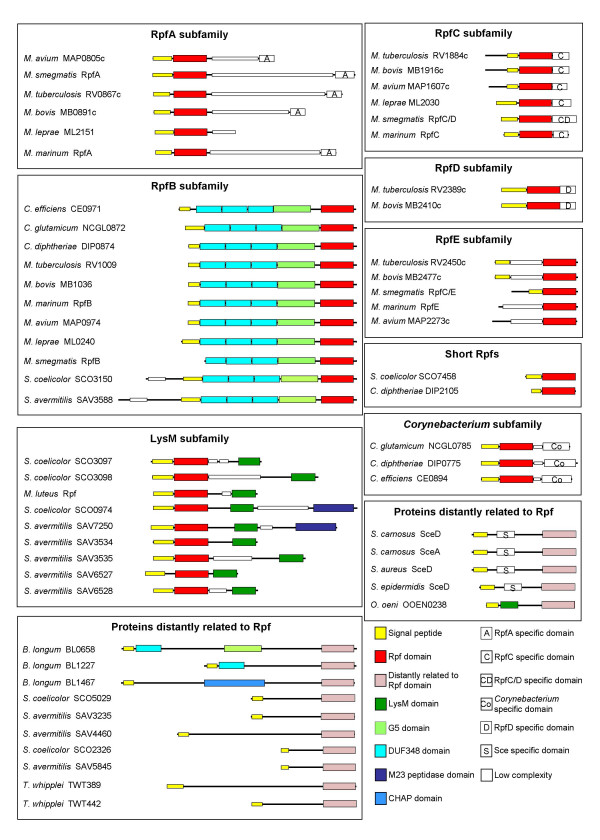
**Domain structure of the Rpf proteins grouped into their subfamilies**. Proteins are from the genomes listed in Table 1. Proteins from organisms whose genome is not yet annotated (*M. marinum *and *M. smegmatis*) have been given the name of the subfamily to which they belong.

Analysis of the various Rpf-like proteins for low complexity regions using SEG, which can separate discrete protein domains [17], and for common motifs using MEME, which can indicate orthologous domains [18,19], indicated that they form ten discrete subfamilies, reflecting their multi-domain architecture. *M. tuberculosis *contains representatives of five of these families, denoted RpfA-E in Fig. 1[7]. A sixth family, containing proteins with the peptidoglycan-binding motif, LysM [20], is restricted to the non-mycolate actinomycetes. A seventh family contains only corynebacterial proteins, while an eighth family contains two short proteins from *Corynebacterium glutamicum *and *Streptomyces coelicolor*, comprising only an Rpf domain.

Proteins more distantly related to Rpf have been grouped together in two additional families. One of these includes the *O. oeni *protein mentioned above; it has an inverse domain organisation compared with that of *M. luteus *Rpf and Rpf-like proteins from *Streptomyces*. The other family of proteins distantly related to Rpf contains two proteins identified following a PSI_BLAST search (3 iterations), using the large N-terminal region of *M. tuberculosis *RpfB (Rv1009) for the first iteration. This protein segment contains three repeats of PFAM-B DUF348 (domain of unknown function) and a G5 domain (also of unknown function, which is found in various proteins involved in cell wall metabolism). The search detected all the previously known RpfB homologues, as well as the two additional gene products from *Bifidobacterium longum *(BL0658 and BL1227; *E*-values 2·10^-59 ^and 9·10^-32^). Several firmicute proteins were also detected (see below). The C-terminal region of the two previously undetected *B. longum *proteins was similar to part (the N-terminal portion) of the Rpf domain (Fig. 1). It was used to search the genpept database downloaded from the National Centre for Biotechnology Information website [21] and this revealed multiple hits in *B. longum*, *Streptomyces avermitilis*, *S. coelicolor *and *Tropheryma whipplei*. The search also detected the *S. carnosus *SceA protein, although this hit was not statistically significant. The actinobacterial gene products detected in these searches are grouped together as a subfamily of proteins distantly related to Rpf in Fig. 1. They were not detected in the original searches using HMMs of the profile of the Rpf domain alignment because similarity with the Rpf domain is restricted to its N-terminal portion (see additional data file 1).

### Proteins similar to RpfB are found in firmicutes

**Figure 2:**
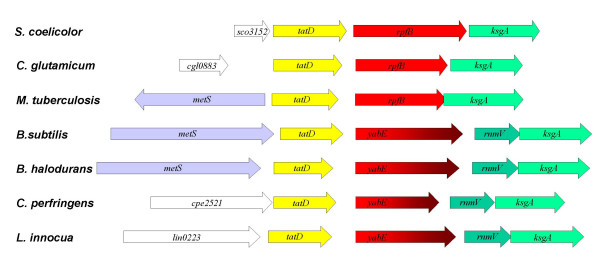
**Genomic context of some *rpfB *and *yabE *genes**. The *sco3152*, *cgl0883*, *cpe2521 *and *lin0223 *genes represented by an empty arrow are hypothetical proteins unrelated to each other. See the text for the designations of the remaining genes.

The link between actinobacterial RpfB and a family of firmicute proteins was noted several years ago, when FASTA was used to search the then available database with Rv1009 (*M. tuberculosis *RpfB) as a query sequence (R. McAdam, personal communication). This detected a *B. subtilis *protein (YabE) of unknown function (23% identity and 38% similarity over 283 residues encompassing the DUF348 repeats and the G5 domain). A HMM model of this protein segment was used to search the TrEMBL and SWISS-PROT databases. In addition to the actinobacterial RpfB proteins, significant hits (*E*-value range 10^-5 ^– 10^-28^) were found to a range of DUF348-containing proteins from various bacilli and clostridia (YabE-like proteins). In these firmicute proteins, the C-terminal Rpf domain is replaced by region of similar size (ca. 60 aa) but totally unrelated sequence. Significantly, *rpfB *and *yabE *(and the gene encoding the distantly related *B. longum *protein) are found in a similar genomic context in the actinobacteria and the firmicutes (Fig. 2).

### YabE is a member of an extended firmicute protein family

A tBLASTN search against the translated GenBank database using the C-terminal segment of YabE as query, revealed similar sequences in more than 40 proteins, suggesting that this is a distinct domain, which we have denoted Sps (**S**tationary **p**hase **s**urvival – see below). This region is also recognized as an uncharacterised conserved domain in the cluster of orthologous groups of proteins COG3584 and has recently been annotated in Pfam (see below). As for the Rpf domain, an HMM profile was created using the newly identified Sps domains and employed to search the TrEMBL database. In addition to the previously identified proteins in bacilli and clostridia, which were detected with highly significant *E*-values (4.4·10^-65 ^– 4.3·10^-35^), these searches also identified some more distantly related proteins with higher, although still significant *E*-values (4.6·10^-7 ^– 2.1·10^-2^). These hits include additional proteins OB0947 from *Oceanobacillus ieheyensis*, CAC2045 from *Clostridium acetobutylicum*, DR0488 from *Deinococcus radiodurans *and TM0568 from *Thermotoga maritima*. The last two hits are the only examples of Sps-like proteins outside the firmicute phylum. Significantly (see below), the CAC2045 gene of *C. acetobutylicum *is annotated as an MltA (membrane-bound lytic transglycosylase A) homologue. Indeed, additional hits above the level of statistical significance in both standard similarity (BLAST) and HMM searches included several lytic transglycosylases from various proteobacteria (see below). Sps proteins are not found in organisms that contain Rpf proteins (Table 2).

**Table 2:** Organisms containing *sps*-like genes

**Part A**: genes encoding proteins containing a Sps domain			
Organism	Genome size (Mb)	No. of genes	Genome Accession Number

*Bacillus anthracis *strain A2012	5.1	5	NC_003995
*Bacillus anthracis *strain Ames	5.2	6	NC_003997
*Bacillus cereus *ATCC 10987	5.2	6	NC_003939
*Bacillus cereus *ATCC 14579	5.4	5	NC_004722
*Bacillus halodurans*	4.2	3	NC-002570
*Bacillus subtilis*	4.2	4	NC_000964
*Oceanobacillus iheyensis*	3.6	4	NC_004193
*Listeria innocua*	3.0	2	NC_003212
*Listeria monocytogenes *EGD-e	2.9	2	NC_003210
*Enterococcus faecalis *V583	3.2	1	NC_004668
*Lactococcus lactis *subsp *lactis*	2.4	1	NC_002662
*Clostridium acetobutylicum*	3.9	2	NC_003030
*Clostridium botulinum *A	3.9	2	NC_003223 (unfinished)
*Clostridium perfringens *str 13	3.0	3	NC_003366
*Clostridium tetani *E88	2.8	2	NC_004557
*Clostridium thermocellum*	3.7	4	AABG03000000
*Desulfitobacterium hafniense*	4.9	1	AAAW00000000
*Thermoanaerobacter tengcongensis*	2.7	1	NC_003869
Phage SPβ*c2*	0.1	1	NC_001884

**Part B**: genes encoding proteins containing a domain distantly related to the Sps domain			

*Oceanobacillus iheyensis*	3.6	1	NC_004193
*Deinococcus radiodurans*	3.1	1	NC_001263, NC_001264
*Thermotoga maritima*	1.9	1	NC_000853

### Sps protein subfamilies

**Figure 3:**
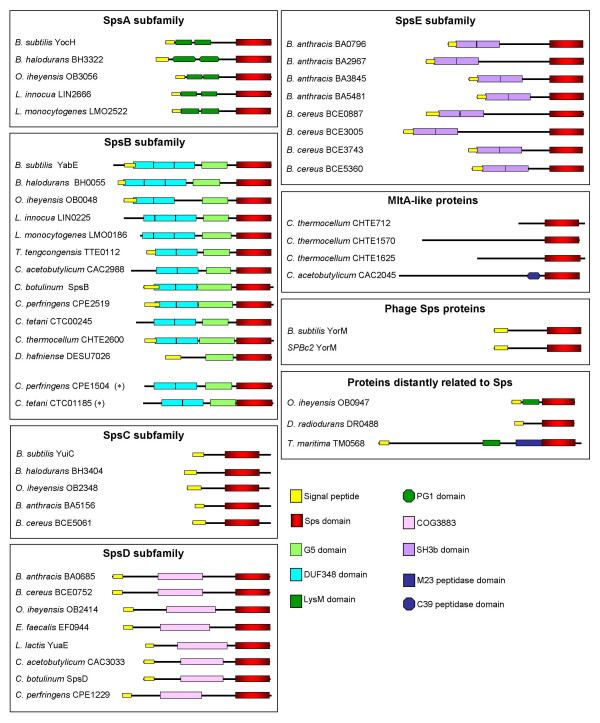
**Domain structure of the Sps proteins grouped into their subfamilies**. Proteins are from the organisms listed in Table 2 with the exception of *B. anthracis *strain A2012 and *B. cereus *ATCC 14579, which contain the same proteins as *B. anthracis *strain Ames and *B. cereus *ATCC 10987 (apart from BA0685 and BCE3743, respectively). Proteins from *C. botulinum*, whose genome is not yet annotated, have been given the name of the subfamily to which they belong. YabE-like clostridial proteins that do not occupy the conserved chromosomal context represented in Figure 2 are indicated with an asterisk.

SignalP [11] and TMHMM [12] predictions suggest that all of the Sps-like gene products so far uncovered are likely to be either secreted, or membrane-associated, with the exception of *Clostridium thermocellum *CHTE712 (Fig. 3). The Sps proteins were also analysed using PFAM [22] and SMART [23,24] for the presence of additional domains. Based on their domain architecture, and the chromosomal context of the encoding genes, they fell into eight subfamilies (Fig. 3). *B. subtilis *contains four genes encoding representatives of four distinct subfamilies. The SpsB subfamily is characterised by the presence of two or three DUF348 domains and a G5 domain, both of which are common to the RpfB subfamily (cf. Fig. 1). The only exceptions are DESU7026 from *Desulfitobacterium hafniense*, which does not have DUF348 domains (but contains a G5 domain and shares the same genomic context as the other members of the SpsB subfamily), together with CPE1504 and CTC01185, from *Clostridium perfringens *and *Clostridium tetani*, respectively. These last two organisms appear to contain two *yabE*-like genes, one in the usual chromosomal context, and another elsewhere (in different positions in the two organisms). The SpsA subfamily is notable as a null mutant of its founder member from *B. subtilis *shows a substantial reduction in post-exponential phase survival (Ravagnani et al, ms. in preparation). These proteins are characterised by the presence of two copies of the peptidoglycan-binding motif LysM [20] (PG1 in the case of *Bacillus halodurans *BH3322), suggesting an association with the cell envelope. Members of the SpsA subfamily do not have a conserved chromosomal context. The other two subfamilies found in *B. subtilis *are the SpsC subfamily, whose members cluster on the basis of their sequence similarity outside the Sps domain and their identical genomic context, and YorM, which is located within the SPβ prophage and is therefore absent from strains that lack this genetic element.

Two more subfamilies not represented in *B. subtilis *are of particular interest as they provided evidence for a link between the Sps proteins and muralytic enzymes. *Bacillus anthracis *and *Bacillus cereus *are the only organisms containing multiple *sps *genes that do not contain members of the *spsB *subfamily. Instead, they have gene products containing two copies of the SH3b domain (SpsE). In bacteria this domain is found in a number of muralytic enzymes, including endopeptidases and amidases. Several Sps proteins from a variety of firmicutes were clustered in another subfamily (SpsD) because they all contain a copy of the putative COG3883 domain. This uncharacterised conserved domain is also shared by a number of muralytic enzymes.

*O. ieheyensis *OB0947, *D. radiodurans *DR0488 and *T. maritima *TM0568 are grouped together because they contain a domain that is only distantly related to the Sps domain (see above). DR0488 is the only known example of an Sps-like protein in an organism with high mole % GC DNA – note however, that *D. radiodurans *is not closely related to the Rpf-containing actinobacteria. The domain structure of TM0568, which has LysM and M23 peptidase domains, in addition to the Sps module, is reminiscent of the Rpf5 proteins from *S. coelicolor *and *S. avermitilis *that contain LysM and M23 peptidase domains in addition to the Rpf module (Fig. 1), and provides another link between these proteins and cell-wall metabolism.

### The MltA-like proteins

**Figure 4:**
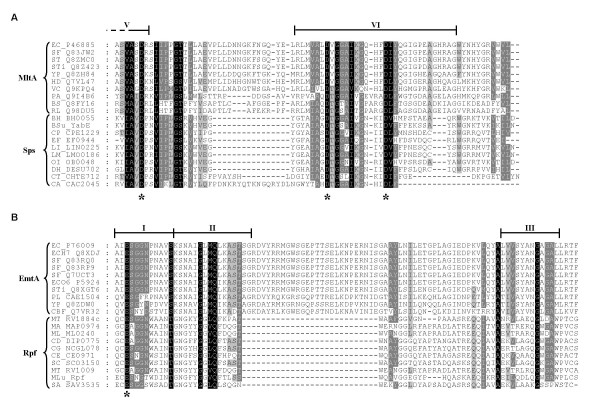
**T-Coffee alignment of MltA & Sps (A) and EmtA & Rpf (B) proteins**. Residues shaded in black are present in 100% of the sequences, dark grey in 80% and light grey in 60%. Bars above the sequences indicate conserved motifs V (partial) & VI from MltA proteins (in Part A) and I, II and III from EmtA proteins (in Part B), as described by Blackburn and Clarke [25]. Putative catalytic residues are marked with an asterisk. Abbreviations are as follows: *B. halodurans *(BH), *B. subtilis *(Bsu), *Brucella suis *(BS), *Candidatus Blochmannia floridanus *(CBF), *C. acetobutylicum *(CA), *C. perfringens *(CP), *C. tetani *(CT), *C. diphtheriae *(CD), *C. efficiens *(CE), *C. glutamicum *(CG), *D. hafniense *(DH), *E. faecalis *(EF), *E. coli *(EC), *E. coli *O157:H7 (ECH7), *E. coli *O6 (ECO6), *Haemophilus ducreyi *(HD), *L. innocua *(LI), *L. monocytogenes *(LM), *M. luteus *(MLu), *M. avium *(MA), *M. leprae *(ML), *M. tuberculosis *(MT), *O. iheyensis *(OI), *Photorhabdus luminescens *(PL), *Pseudomonas aeruginosa *(PA), *Rhizobium loti *(RL), *Salmonella typhi *(Sti), *Salmonella typhimurium *(ST), *Shigella flexneri *(SF), *S. avermitilis *(SA), *S. coelicolor *(SC) and *Yersinia pestis *(YP).

Three proteins from *Clostridium thermocellum *and one from *Clostridium acetobutylicum *represent the eighth subfamily of Sps proteins (Fig. 3). In these proteins, the Sps domain overlaps with a region of strong similarity to the Gram-negative membrane-bound lytic transglycosylase, MltA (Pfam *E*-value = 10^-6 ^– 10^-7^). Indeed, Pfam predicted potential matches with MltA for all the Sps proteins, although with lower *E*-values (10^-2 ^– 10^-3^). HMM profiles were built from the known lytic transglycosylases using the classification proposed by Blackburn and Clarke [25]. Local and global searches of the *B. subtilis *genome using these profiles detected two known and six new putative lytic transglycosylases. Five of these (YjbJ, YomI, YqbO, YddH and YkdO) were similar to the family 1 of goose-type lysozymes. The remaining three, which are similar to the MltA-type family 2, are the Sps proteins, YocH, YuiC and YabE (*E*-values in local searches 4.1·10^-5^, 5.6·10^-6 ^& 2.3·10^-2^, respectively). The fourth *B. subtilis *Sps protein, YorM, which lies within the SPβ prophage, was not detected. Blackburn and Clarke [25] distinguish six motifs within the MltA-type family 2 consensus sequence. The Sps domain encompasses motif 6 and part of motif 5. This region contains three conserved aspartate residues that may be involved in catalysis [25]. Significantly, these residues are absolutely conserved amongst all the 46 known Sps domains (Fig. 4A) as recently recognised in Pfam (3D domain).

These observations acquire even greater significance in the light of the weak similarity that has been noted between the Rpf domain and the goose-type lysozymes [2,14,16]. Blackburn and Clarke [25] identified four motifs in the consensus sequence of this type of lytic transglycosylase, and divided the family into five subclasses according to two more variable motifs 3 and 4. The C-terminus of the Rpf domain encompasses motifs 1 and 2 of the EmtA-type family 1e, which includes the absolutely conserved catalytic glutamyl residue (Fig. 4B).

## Discussion

**Figure 5:**
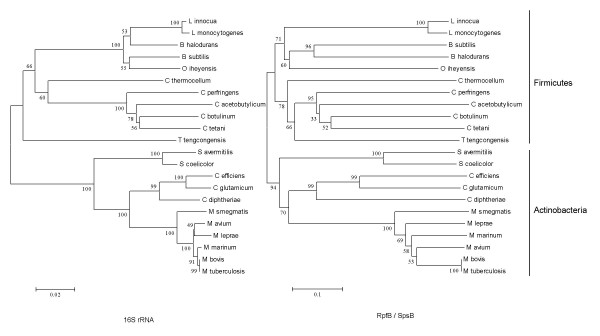
**Phylogenetic analysis of the RpfB and SpsB proteins**. Phylogenetic trees based on the N-terminal moieties (DUF348 & G5 domains) from RpfB and SpsB proteins (right) and 16S rRNA sequences of organisms that contain RpfB and SpsB proteins (left). Trees were constructed by neighbour joining methods using MEGA v2.1 [77]. Bootstrap values are shown at the branch points.

We have presented evidence indicating that the firmicutes contain a family of proteins functionally equivalent to the actinobacterial Rpf family. The original link between the two protein families was provided by *M. tuberculosis *RpfB and *B. subtilis *YabE, which share a large N-terminal region containing DUF348 and G5 domains. In spite of this striking similarity, YabE lacks a C-terminal Rpf domain and contains instead a domain of similar size that we have called Sps (see above). Although the Rpf and Sps domains are totally unrelated in both sequence and secondary structure (see additional data files 1 and 2), we have presented evidence that they have a similar biological function. According to the definition proposed by Koonin *et al*. [26], an event of non-orthologous gene displacement can be suspected when the same function is fulfilled by unrelated or distantly related proteins. The RpfB and YabE proteins provide an example of a related phenomenon applicable to protein domains that we have called "non-orthologous domain displacement". Phylogenetic trees constructed using only the shared N-terminal region of RpfB-like and YabE-like (SpsB) proteins (Fig. 5) resemble trees generated with 16S rRNA, suggesting that these proteins have undergone vertical transmission from a common ancestor and that the Rpf domain displaced the Sps domain (or *vice versa*) sometime after the actinobacterial and firmicute lineages diverged. Most probably, this event has been followed by duplication and diversification within each lineage to create paralogues of the Rpf proteins in the actinobacteria and the Sps proteins in the firmicutes. Other instances of what could be referred to as non-orthologous domain displacement have been documented previously, e.g. aminoacyl tRNA synthetases. Bacterial and eukaryotic glutamyl-tRNA synthetases have generally similar domain architectures but they contain unrelated anticodon-binding domains [27,28]. Similarly, eukaryotic tyrosyl tRNA synthetases contain two domains that are unrelated to those of their bacterial counterparts [28,29]. The DnaG-like primases of bacteria and their phages differ from their archaeal orthologues in that the former contain a Zn-finger DNA-binding domain, whereas the latter contain a helicase-derived domain probably involved in the same function [30,31]. Protein domains are considered as the basic units of folding, function and evolution [32-35] and we suspect that the phenomenon of non-orthologous domain displacement could be quite widespread. Moreover, it might have predictive value in cases where the function of only one of a pair of non-orthologous domains is already known.

Most *rpfB *and *spsB *genes lie within a very similar genomic context flanked by *tatD *and *ksgA*(with *rnmV *inserted between *spsB *and *ksgA *in firmicutes). The only exceptions are the duplicate *spsB *genes found in *C. perfringens *and *C. tetani*, one of which is located elsewhere in both organisms. Statistical analysis of the enormous amount of genome sequence information that has become available in recent years has shown that conservation of genome context may often be employed to infer functional relationships between neighbouring genes [36]. In our case, a functional association is indeed predicted by the SNAP algorithm (Similarity Neighbourhood APproach [37,38]), though it is not obvious what the relationship might be. TatD is a Mg^2+^-dependent deoxyribonuclease of unknown function [39], RnmV is a ribonuclease M5/primase-related protein involved in maturation of the 5S rRNA [40,41] and KsgA is a 16S rRNA methyltransferase that may play a role in translation initiation [42]. In *B. subtilis *the *tatD *(*yabD*) gene does not appear to be expressed during either vegetative growth or sporulation, whereas the *rnmV *(*yabF*) and *ksgA *genes appear to be co-transcribed during vegetative growth. They are highly expressed at the beginning of exponential phase and their expression declines sharply shortly afterwards, an almost identical pattern to that of *yabE *(data from the *B. subtilis *Genome Database [43]. These observations may reflect a connection between protein synthesis (RnmV, KsgA) and cell wall expansion (RpfB or SpsB – see below) as would be required when a cell restarts growth after dormancy (in the case of Rpf) or prolonged stationary phase (in the case of Sps). The SNAP algorithm also predicts a functional association between RpfB/SpsB and the 4-diphosphocytidyl-2C-methyl-D-erythritol kinase. The gene encoding this protein (*ispE*) is located immediately downstream of *ksgA *in actinobacteria and two to four genes downstream of *ksgA *in *Listeria *and *Bacillus *spp., respectively (however, it appears to have a scattered distribution in clostridia). The 4-diphosphocytidyl-2C-methyl-D-erythritol kinase participates in the non-mevalonate pathway for isoprenoid synthesis, which is involved in cell wall biosynthesis in *E. coli *and *B. subtilis *[44].

A functional relationship between neighbouring genes is normally inferred when they also show the same phylogenetic profile. This is not universally true in the present case, since some firmicutes, e.g. *S. aureus*, *Streptococcus agalactiae*, *Streptococcus pyogenes*, *B. anthracis *and *B. cereus*, contain neither *rpfB *nor *spsB *although the other genes normally associated with them, *tatD*, *ksgA *and *rnmV *(in firmicutes) are present in the same relative order. Presumably, *rpfB *or *yabE *have been lost from these organisms (the alternative, necessitating several independent gene acquisition events, seems less likely). This is particularly evident in the mollicutes, where the occurrence of the genes in question is patchy. None of the strains sequenced contain *rpfB*/*spsB *(these organisms lack a cell wall), but some contain *rnmV-ksgA *(*Mycoplasma capricolum *and *Mycoplasma mycoydes *– D14983 and NC_005364, respectively), some contain *tatD-ksgA *(*Mycoplasma pulmonis*, NC_002771) and some contain only *ksgA *(*Mycoplasma genitalium*, *Mycoplasma gallisepticum*, *Mycoplasma penetrans *and *Mycoplasma pneumoniae *– NC_000908, NC_004829, NC_004432 and NC_000912, respectively). As mollicutes are believed to derive from bacilli by reductive evolution [45], it seems that this group has lost *rpfB*/*spsB *and is in the process of loosing the remaining genes in the string. Note that *rpfB*, *yabE *and *ksgA *are non-essential genes [6,8,46] (Ravagnani et al., in preparation), as are *tatD *and *rnmV *in *B. subtilis *[41,43]).

Information from gene fusions may also be used to predict gene function. The "Rosetta stone" [47] and "guilt by association" [48] approaches propose that if a combination of domains A and B is detected in one protein and a combination of domains B and C in another, then it may be predicted that domains A, B and C are functionally related. The "Rosetta stone" hypothesis suggests that the function of one protein domain may be predicted on the basis of its fusion to another domain of known function. Since we do not know the function of the domains connecting RpfB and SpsB (DUF348 & G5), it might be more correct to invoke "guilt by association" in the present case.

More recently, a new method based on consideration of genomic context has been employed to predict orthologous relationships between genes on the basis of anti-correlating occurrences of genes across species [49]. Given three genes A, B and C, if A is always present in a particular group of organisms in association with either B or C, but B and C are never found in the same organism, it can be predicted that B and C fulfil the same function. Extending this approach to protein domains, we may predict that the Rpf domain of RpfB and the Sps domain of SpsB have the same function, as they are both fused to the same DUF348- and G5-containing region, but never occur in the same organism (or, at least, in those so far sequenced).

In bacteria, the DUF348 domain appears to be restricted to proteins containing either Rpf or Sps domains (but it is also found in the yeast Myb-like protein Snt1). *B. anthracis *and *B. cereus *are the only organisms containing multiple *sps *genes that do not have an *spsB *gene, despite conservation of the genes with which it is normally associated (*tatD*, *rnmV *and *ksgA*). These bacteria have instead four and three copies, respectively, of *spsE *genes encoding proteins containing two SH3b domains. SH3b is the equivalent of the eukaryotic SH3 (Src homology 3) domain, which is found in a variety of membrane-associated and cytoskeletal proteins and mediates protein-protein interactions by typically binding proline-rich polypeptides [50]. In bacteria, SH3b domains are found in various cell wall amidases and peptidases. Although their function is unknown, the SH3b-containing region of *Staphylococcus simulans *lysostaphin, which cleaves peptidoglycan, mediates binding to the *S. aureus *cell wall [51]. Such a function would be consistent with the occurrence of this domain in muralytic enzymes. It is tempting to suggest that the DUF348 domain has a role similar to that of the SH3b domain. Whatever their functions might be, invoking again the principle of "guilt by association" [48], the association of the Sps domains with other domains present in muralytic enzymes (SH3b, COG3883, LysM) points very strongly to a role for the Sps proteins in cell wall metabolism. This hypothesis is also supported by the occurrence of an M23 peptidase domain in *S. coelicolor *and *S. avermitilis *Rpf5, *Thermotoga maritima *TM0568 and some lytic transglycosylases, such as *B. subtilis *YomI.

The sequence similarity between the C-terminal region of the Sps domain and that of the Gram-negative membrane-bound lytic transglycosylase, MltA, serves to reinforce this connection. Figure 4A shows that the similarity between Sps and MltA encompasses all three aspartate residues that have been highlighted as potential catalytic residues for the lytic transglycosylase family 2 – classification according to Blackburn and Clarke [25]. In parallel with this, there is also sequence similarity between the Rpf domain and the N-terminal region of the Gram-negative endo membrane-bound lytic transglycosylase, EmtA [2,14]. Although quite limited, the similarity in this case encompasses the absolutely conserved catalytic glutamate residue of the lytic transglycosylase family 1 (Fig. 4B).

Lytic transglycosylases are enzymes that catalyse cleavage of the β-1,4-glycosidic bond between N-acetylmuramic acid and N-acetylglucosamine in the peptidoglycan backbone. Unlike lysozyme, they also catalyse an intramolecular glycosyltransferase reaction to form terminal 1,6-anhydromuramic acid-containing products. The exact function of these enzymes is unknown, but they are thought to be involved in cleavage of the peptidoglycan to permit the insertion of newly synthesised material during cell elongation and division. Remodelling of the cell envelope requires the concerted action of both hydrolases and synthetases, which may form large multienzyme complexes [52,53]. Consistent with this, physical interactions between some *E. coli *lytic transglycosylases and penicillin-binding proteins (enzymes involved in the synthesis of peptidoglycan) have been demonstrated experimentally [54,55].

In *E. coli *there are at least six lytic transglycosylases, one soluble and five membrane-bound [56-60], with different substrate specificities. Due to the high degree of redundancy, no obvious effect on growth is observed after deletion of their genes [60]. This is in agreement with the results obtained after disruption of three of the five *rpf*-like genes in *S. coelicolor *[2] and the five *rpf*-like genes of *M. tuberculosis *[6,8]. In contrast, there is evidence for essentiality of the apparently unique *rpf *gene of *M. luteus*, whose chromosomal copy could be disrupted only in the presence of an extra plasmid-encoded copy of the gene [5]. However, definitive proof of essentiality would require the construction of a conditional mutant and this technology is not currently available for *M. luteus*.

In *B. subtilis *the *sps *genes are not essential, but a clear phenotype is associated with disruption of *yocH *and this is much accentuated by the disruption of all four *sps *genes: these mutants show reduced survival after prolonged stationary phase (Ravagnani et al., ms. in preparation). This phenotype has been observed previously, associated with disruption of genes involved in cell wall metabolism, such as the *E. coli nlpD*, encoding an M23 endopeptidase [61], and *surA*, encoding a peptidyl-prolyl isomerase [62]. The latter is required for the correct folding of extracytoplasmic proteins and it has been proposed to be necessary for the assembly of the murein-synthesizing complex, of which lytic transglycosylases are a component [62]. In the Gram-positive bacteria, *rpfB *or *spsB *occupy a highly conserved genomic context, within a group of genes including *ksgA *(see above). Interestingly, in *E. coli *and related enteric bacteria, *ksgA *lies within the same transcription unit as *surA *(*surA-pdxA-ksgA*-*apaG-apaH*), suggesting again a possible association between protein synthesis and cell wall expansion.

The assignment of a muralytic function to the Sps and Rpf domains is entirely consistent with the presence of an Sps protein, YorM, in the *B. subtilis *prophage SPβ, and the recent discovery of the Rpf domain in a large mycobacteriophage "tape measure protein" [13]. Muralytic transglycosylase activity is often associated with bacteriophage virions and confers upon them the highly localised muralytic activity that is required for the process of phage infection, without provoking premature lysis of the host [63].

The bioinformatic evidence in favour a role for the Rpf and Sps proteins in peptidoglycan metabolism is now compelling. This prediction has recently been confirmed; both *M. luteus *Rpf and *B. subtilis *YocH have murein hydrolase activity in zymograms (Mukamolova et al., ms. in preparation; Ravagnani et al., ms. in preparation).

## Conclusions

As a result of the observed catalytic activity of the Sps and Rpf proteins, our views on the nature of bacterial non-culturability are changing. The various models of non-culturability we have developed over the years [1,64,65] might be explained by the disappearance of nascent peptidoglycan and its gradual replacement by inert peptidoglycan in the bacterial cell wall. This has recently been proposed as a key feature of the mechanism that determines the position of growth zones in the bacterial cell wall [66-68]. We suggest that the walls of non-culturable organisms may contain such a preponderance of inert peptidoglycan that their envelope has effectively become a "cocoon", requiring the action of specialised muralytic enzymes to make a restricted number of scissions, before growth and wall expansion can resume. The Sps and Rpf proteins may have been recruited to serve this function. Resumption of cell wall synthesis might therefore be regarded as one of the "core processes" (see above), along with re-initiation of protein synthesis, that would need to be activated by cells emerging from dormancy (in the case of Rpf) or prolonged stationary phase (in the case of Sps). Signalling could be part of such a resuscitation mechanism, mediated perhaps by a small molecule released from murein as a result of the action of Rpf / Sps proteins. This hypothesis is currently being tested.

## Methods

Database searching was carried out using either the position-specific iterative BLAST (PSI-BLAST) method [69] or the Hidden Markov model (HMM) database searching algorithm of HMMER 2.2 g . Both local and global profiles of aligned sequences were generated, and searches were carried out using the default parameters. For one application, FASTA [70] was employed.

Domain analysis was undertaken using COG [71-73], MEME [18,19], PFAM [22], SEG [17], SignalP [11], TMHMM [12] and SMART [23,24].

Sequence alignments were generated using ClustalX version 1.81 [74] and T-coffee [75,76].

Phylogenetic trees were generated using MEGA v2.1 [77]. T-coffee-aligned sequences were analysed using the neighbour-joining method (options: p-distance model, compete removal of gaps, 10,000 bootstrap replications).

## Authors' contributions

AR carried out the bioinformatic analysis of the Sps proteins and drafted the manuscript. CLF carried out the bioinformatic analysis of the Rpf proteins. MY supervised the project and contributed to drafting of the manuscript. All authors read and approved the final manuscript.

## Supplementary Material

Additional File 1 **Sequence alignment of the Rpf domains **Clustal X alignment of Rpf domains (A) and of domains distantly related to the Rpf domain (B). Click here for file

Additional File 2 **Sequence alignment of the Sps domains **Clustal X alignment of Sps domains (A) and of domains distantly related to the Sps domain (B). Click here for file
