# Supplementary material for: A novel firmicute protein family related to the actinobacterial resuscitation-promoting factors by non-orthologous domain displacement
Source: BMC Genomics. 2005 Mar 17;6:39. doi: 10.1186/1471-2164-6-39 (PMC1084345; doi:10.1186/1471-2164-6-39)
Supplement: Additional File 1 — Sequence alignment of the Rpf domains Clustal X alignment of Rpf domains (A) and of domains distantly related to the Rpf domain (B). [file 1471-2164-6-39-S1.doc]

# A

Jpred : -HHHHHH-----------------E-----HHH-------------HHHHHHHHHHHHHH--------

*****

## **B**

*****
