# Supplementary material for: A novel firmicute protein family related to the actinobacterial resuscitation-promoting factors by non-orthologous domain displacement
Source: BMC Genomics. 2005 Mar 17;6:39. doi: 10.1186/1471-2164-6-39 (PMC1084345; doi:10.1186/1471-2164-6-39)
Supplement: Additional File 2 — Sequence alignment of the Sps domains Clustal X alignment of Sps domains (A) and of domains distantly related to the Sps domain (B). [file 1471-2164-6-39-S2.doc]

# A

*****

*****

*****

jpred : --EEEEEEE--------------------E--------EEE--------EEEEEE---------EEEE----------EEEEE---------EEEEE----HHHH------EEEEEE---

## **B**

*****

*****

*****

**Figure 2S** Clustal X alignment of Sps domains (A) and of domains distantly related to the Sps domain (B). Residues shaded in black are present in 100% of the sequences, dark grey in 80% and light grey in 60%. Putative catalytic residues are marked with an asterisk. Abbreviations are as follows: *B. anthracis* str Ames (BA), *B. cereus* ATCC 10987 (BC), *B. halodurans* (BH) *B. subtilis* (BS), *C. acetobutylicum* (CA), *C. botulinum* (CB), *C. perfringens* (CP), *C. tetani* (CT), *C. thermocellum* (CTh), *D. radiodurans* (DR), *D. hafniense* (DH), *E. faecalis* (EF), *L. lactis* (LL), *L. innocua* (LI), *L. monocytogenes* (LM), *O. iheyensis* (OI), Sps consensus sequence (Sps_CON), *T. tengcongensis* (TT), *T. maritima* (TM). The secondary structure prediction shown in part A (bottom) was generated using Jpred at <http://www.compbio.dundee.ac.uk/~www-jpred/>. (H) alpha helix; (E) extended beta sheet.
